# Awoke in His Coffin

**Published:** 1886-07

**Authors:** 


**Awoke in His Coffin.**—A singular story comes to us from Clinton, Ky. George O. Daniels, of that place, had been ill for several months, and last Wednesday, to all appearances, died. The remains were put in a casket, where they remained for twenty hours awaiting the arrival of relatives to attend the funeral. At midnight the watchers were startled by a deep groan emanating from it, and all but one rushed from the room. He remained, and as the groans con-continued, raised the coffin lid and saw that Daniels was alive. Seizing the body he placed it upright. A few spasmodic gasps, a shudder and the corpse spoke. The relatives returned to find the man sitting in a chair and conversing with reasonable strength. Mr. Daniels claims to have been perfectly conscious of everything passing around him, but says he was unable to move. He heard the sobs of his relatives when pronounced dead by the doctors, and noticed the preparations for the funeral. He is about eighty years of age.

**The July Century** comes to us as usual, laden with good things. It has come to be par excellence, the record of militay events of the late civil war, the writers being for the most part, northern and southern officers who took part in the conflict. Among the articles of interest in the present number, are "Fighting Farragut below New Orleans," "Incidents of the occupation ot New Orleans," and "Cross Country Riding in America." We commend this magazine as one of the very best of its kind.

### HOME DEPARTMENT.

**Potato Gems.**—One warm potato, mashed fine, soften with tepid water, then stir in Graham flour, unsifted, until a gem-batter is formed. Beat well, drop into hot gem pans, bake in a good even oven thirty to forty minutes.

**Corn Dodgers.**—Mix corn meal with cold water, making dough stiff enough to handle. Then mould into oval cakes about two inches thick, put them in an oiled pan and smooth the top with the hand wet with cold water. Bake in a hot oven forty or fifty minutes.

**Potato Puffs.**—Take an egg, one cup of cream or milk, two cups cold or hot potato ; this must be mashed till there are no lumps. Beat the egg, stir the milk or cream into it, and then add the potato; beat all together, pour into an oiled pudding dish, bake in a quick oven till the top is nicely browned.

**Hygiene Rusk Crums.**—Take bits of unleavened wheaten bread, dry them thoroughly in an oven hot enough to brown slightly, but not scorch. Break them in a mortar and grind in a coffee mill. Or take a stale graham loaf, grate it, brown in the oven and when brittle roll fine. Serve with fruit juice or milk, allowing it to soak a few minutes before it is eaten. Parched wheat may be ground and eaten in the same way.

**Summer** is the most trying time for babies, and as we are on the subject another suggestion is offered. The prevailing troubles at this season are caused by overheating. Now a cradle can be made cool, protecting, comfortable, by having a framework made of the shape of an ordinary cradle, and then covering it on all sides, top and bottom as well, with wire gauze. The top should have a lid which will shut down, and the whole thing can be put upon rockers as with any cradle. It is not complicated, and any good carpenter can easily make one from this description for the cost of an ordinary cradle. With the lid let down no fear of flies or bugs need be felt, and the baby needs no mass of heated clothes over or under him. Neither is it close or uncomfortable.

**When Tea is Harmful.**—There is a curious observation which has occurred to me in connection with the disturbing influence of tea on digestion. You will find that certain persons can at certain times take tea in moderation without its producing any disturbing effect on their digestive processes, while at other times its use leads to much distress and discomfort, causing annoying palpitation and an uneasy sensation almost amounting to actual pain about the upper part of the cardiac region, generally on the left side. Now you will find that this difference in their capability of tolerating tea is often dependent on some emotional disturbance of the nervous system ; the instant there is any worry or anxiety or mental uneasiness tea will at once disagree with such persons, and when the emotional wave has passed by, and a condition of nervous calm has been reestablished, the use of tea can be resumed. It is often important to be able to recognize this disturbing influence of tea and coffee, especially on cardiac innervation, and particularly in the production of that peculiar præcordial pain which I have described above, and to which I am not aware that attention has been previously called.—*Dr. Yeo.*

There is nothing truer than the well-attested fact that tea is not, as a rule, a healthy drink, that it is always more or less deranging to the action of the stomach. When to this fact is added the one important one, which is well ascertained, that not one pound in ten thousand is genuine tea and tea only, it is far better to let it entirely alone and to drink chocolate or water, or, still better, drink nothing at all with our meals.

### WHAT IT COSTS AND WHAT IT SHOULD COST.

The cost of living has been one of the great questions prominently brought forward by the late strikes and agitation over the labor problem during the last few months, and what is a little singular, nothing or very little has been said about it either as an economical necessity or an extravagant luxury. It is our province to look squarely in the face of this question and to speak of the facts as they exist.

The great fact is that at the present prices of labor, a man who has a large family, who attends to them properly, who does not spend his time at the grog-shop and who does not allow his home to be made a grog-shop ; lives comfortably, his children are well dressed, his wife neat, her home always inviting, with an air of comfort surrounding everything connected with it. On the other hand, as we have seen in a manufacturing village, his neighbor, getting the same wages, is unhappy in his family and his home inside and out looks like a resort more for his pigs than for his children.

Can you tell what makes the difference ? One pays no more for the doctor than the other, no more for his eatables, and his wife and children are not extravagant. Where has the money gone ? He has not spent it for the general use of his family. Look about you as you pass his residence Beer bottles and whiskey bottles by the dozen, and they are only the small portion he has brought home ; the keeper of the beer-shop has a much larger portion. Here is the source of nearly all the rioting and the striking in the Country. We do not say that it is the only cause, for in many instances the demand for better pay is just ; but the method adopted to get it was a mistake, to say the least.

But let us look at the way money is spent for living in this land of laborers, among those who do the labor. One of the greatest of errors among this class, or among all classes of Americans, is the popular belief that meat is the most nutritious food that can be obtained, and meat must be had although its cost is greater than more nutritious diet.

Let us look, for instance, at roast beef. It costs anywhere from 12 to 20 cents per pound. For ten cents a piece of beef can be purchased that would make seven pints of beef soup fully as nutritious and strength-giving as a seven pound roast. This is making a better diet than meat, at a much cheaper rate—all the virtue is in the soup without waste. Of course, there are plenty of people who will exclaim: "Oh, I can't eat eat soup! I don't like soup; never did and never can!" We have seen whole families who never touched soup, and one of the reasons was because they never had a bowl of soup that was fit to eat. Soup is a dish fit for a king if it is properly made, but if made up in the regular slop way of Americans generally, it is wholly unfit for human kind.

How would we make it? Well, first, have the materials to make it of. It does not matter what the soup is to be made of so much as how it is made. Whether beef, veal, mutton or chickens, the meat should first be cleaned and washed. Next, put it into a kettle and cover with cold water. As it comes to a boil skim it off as fast as the floating material rises. When it has boiled three minutes, wash it in boiling water, turn off all the water and rinse the kettle. Put on the meat in clean boiling water, (having the tea kettle or other vessel full of boiling water for this purpose), and let it have a steady boiling of three hours. During the last half-hour crusts of toast-bread may be added, or a handful of rice, or grits, or if it is desirable parsley may be added in the second water. It should not have the salt or pepper added until it is taken from the stove, and it is preferable to have each one season it as the taste may be inclined.

This constitutes a soup that is palatable and nourishing. This is a soup that stays by a man to do a day's work, and with the addition of some hard bread, butter and a dish of sauce, or oat-meal and milk (not all mixed together but eaten seperately or at most two or three articles eaten with the soup), a man can do more effective laborious work and retain better health than upon a diet of heavier food. And if for supper, instead of the eternal filthy cup of tea, the soap baking powder biscuit, the hog-fat cake covered with a sulphuric acid sugar, and an alum curd called a jam, the good industrious people would eat plain bread buttered, or oat-meal mush, corn-meal mush with milk or honey and a drink of cold or hot water, not alone could they save money but they could save doctors' bills and waste of time.

The appetite for solid meat, as baked meats, roast pork, roast beef, &c., &c., is acquired, but it is not necessary to health or strength. The stoutest races of the world are oat-meal eaters. We are not advocating an entirely vegetarian diet, but we advocate a much less indulgence in meats, baked, roasted or boiled, if one wants to regain their health and fill up the pocket-book. The idea of a meat diet being a prime and vital necessity is absurd. We eat too much meat for health, and we burn the laborers' wages up when we attempt to build up a muscular structure entirely or principally on a meat diet.

We have known the hardiest men who have seldom touched meat, but who drank the water from oat-meal as a constant drink. Three quarts oat-meal put into a bucket and filled with cold water, when it settled is was drank freely while laboring at the heaviest of tasks. The idea that one has to have a pound or two of beef to eat is as absurd as the idea that he has to have from three to five glasses of beer to drink. The most solid flesh is built up of vegetables. The clearest brains are those who are nourished on the farinaceous grains. And as for money—well we say deliberately we cannot believe that a laborer can save money when his wife pays out a days wages for one or two meals of meat. Perhaps this ought to come under the head of the Great Economies, but usually it is a leak that very few look into, but when once seen through, either by the master or mistress, a great leak is stopped in the expenses of the household.

**Whooping Cough.**—The following is given as an improvement on sending children to the gas works as a remedy for whooping cough. Attach a piece of rubber tubing to a gas burner, the tubing being long enough to reach the floor. Let the child inhale it sparingly and at intervals, for a few minutes at a time. This is probably good treatment for those who live within the gas using districts.

**The Bromide of Camphor** is highly spoken of in the treatment of chorea in children. It is best given in capsules.

### MISCELLANEOUS ITEMS.

**Hereclides** was an empiric.

**Esculapius** was the founder of Greek medicine.

**Melampices** was the first Greek physician who was known to the world.

**Asclepiades** practiced medicine in Rome 90 years before the birth of Christ.

**Tincture of Gelseminum** is mentioned as very beneficial in nervous deafness, in small doses often repeated.

**Mustard** mixed with white of egg, is said to produce all the good effects of mustard without blistering.

**Cold Water** and opium are great remedies for wounds, says Dr. W. J. Harris, and we say that, in many cases, cold water alone is the best remedy.

**Claudius Galenus** was the name of a famous physician who was born A. D. 130, and studied under Pelodes, and his cyclopedia of medicine was a text book till the 14th century.

**One** of the best possible applications for a burn, and one recognized by most medical authorities as one of the best articles which can be used, is common white paint (carbonate of lead).

**Honey** is recommended by a writer in the Lancet, as one of the best disguises of the taste of quinine, and especially valuable to this end when administering the drug to children. The dose should be placed in the centre of a teaspoonful of honey.

**Hall's Journal of Health** advertises no remedy which is not understood to be harmless in use and effectual in operation, and any medicine advertised in its columns when ordered through its business department, will be procured and sent at the wholesale price.

**Sir Henry Thompson** holds that artificial teeth are an evil in those advanced in years, because they enable such persons to chew flesh. He considers that when teeth decay and fall out it is nature's intention that man should live on a vegetable diet. Whether that is the reason for decaying teeth is extremely doubtful, but about the eating of animal food there is no room for there being any doubt whatever.

**Beef Tea** has less nutriment in it than eggs or milk, and is not near so good a food for the sick.

**Be careful** this hot weather, and do not expose your bodies to drafts when in free perspiration.

**Ventilate,** *ventilate,* VENTILATE. This is the first and great commandment of good health and sanitary science, and the second is like unto it.—Keep clean in body and in clothing.

**In case of poisoning** by poisonous plants, as poison ivy, poison oak, etc., apply frequently to the affected part a solution made up of one dram each of bicarbonate of soda and sulphite of soda to one ounce of water.

**The Heat.**—Now is the time of summer complaint from extreme heat and carelessness of diet. Sunstroke, heat fever, some skin affections, and some diarrhœas and summer complaints. Avoid heat exposure and these consequent diseases. Aside from the action of heat in producing cholera infantum, dysentery, etc., the worst forms of these diseases come from fermentation and germs introduced into the body by eating sour or unfit food, or green fruit.

**Wanting to Learn.**—All these people, (Hindoos, Chinese and Jews), are very glad to sit at the feet of Western Medicine and learn. They clamor for our schools, and our treatment, and surgery, which is as much an advance over their own as the electric light is over their own olive oil lamps.—*Albany Sunday World.*

That's true and we are happy to see the change and the desire to learn now. There is nothing more needed at the present time among all these people, than a more complete knowledge of surgery and medicine.

**Creosote a Specific for Erysipelas.**—Dr. J. H. Cox gives some experience of his own in this remedy, and says. "All injuries, of whatever kind, have been treated with dressings of this remedy, and where this has been done from first to last, in no instance has there been an attack of erysipelas."

"The usual manner of application, was in solution, of six to twenty drops, to the ounce of water, keeping the parts covered with clothes constantly wet with it. In ulcers or wounds it may be used in the form of a poultice, by stirring ground slippery elm into the solution. The strength to be regulated according to the virulence of the attack. Ordinarily, ten drops to the ounce is strong enough for the cutaneous form of the disease, and in dressings for wounds or recent injuries. If the inflammation threatens to spread rapidly, it should be increased to twenty or more drops to the ounce of water.

**Give Infants Water.**—Babyhood sagely says, "Only those who watch infants with intelligent discrimination know how often they suffer from fever. With this fever comes thirst. What does the mother put into that little dry mouth? Often nothing but milk! When we adults have fever do we find that milk relieves the thirst? Does it not rather increase it? Be assured, it is the same with the baby. With the slightest symptoms of fever, cold water administered with a teaspoon is the prescription of wisdom and mercy.

**A Remedy for Hiccough.**—Dr. A. G. Gibson calls attention in the *Edinburgh Med. Journ.*, April, 1886, to the old Hippocratic aphorism, "Sneezing occurring after hiccough removes the hiccough," and suggests, in cases of hiccough, the production of sneezing by tickling the nostrils, and he tells us that he has in this way been very successful in arresting this disagreeable affection. Hiccough, as well as sneezing, is one of the specially modified respiratory movements, and it is quite in accordance with what we know of the transference of nervous action, that the spasmodic contractions of the diaphragm should cease on the induction of the explosive expirations which constitute the acts of sneezing. There is one point, however, which deserves special mention. It is not necessary that the stimulus applied to the nose be followed by sneezing. The application of a gentle irritant to the nasal mucous membrane may be quite enough to put a stop to the hiccough, by diverting the nervous energy into other channels, although it may not be of sufficient power to induce sneezing.

**Employment of the Insane**—At last, we have a movement in the management of the insane which shows some signs of beneficial results Dr. C. R. Agnew writes as follows. "Two months ago the managers of the Hudson River State Hospital for the Insane, Poughkeepsie, N. Y, established a day school for teaching the branches of a common school education to the patients under their care. It was done in the hope of affording a diversion for the patients and as a palliative for the monotony of asylum or hospital life. In the school, which is under the care of a competent male teacher, there are classes for men in the morning and for women in the afternoon. Reading, writing, arithmetic, spelling and singing are taught. At my last visit of inspection as manager, I saw eighty men engaged in a contest in spelling, and the effect of the healthful exercise of the mind of the patients was to me most promising. All who are in the management of hospitals for the insane know how difficult it is to plan for a daily life for the patients in which shall be provided a due allotment of such mental work as the latter are individually capable of doing. Frederick Peterson, M. D., the first assistant physician of the hospital, has kindly furnished notes of three cases, which may illustrate the progress of the work. Perhaps our experiment at Poughkeepsie may open a useful line of inquiry and experience."

